# Allium covered metal stent for treatment of malignant ureteral obstruction

**DOI:** 10.3389/fsurg.2024.1445808

**Published:** 2024-11-19

**Authors:** Jing Qing, Ke Hu, Xuan Zhang, Huaming Luo, Jiangchuan Chen, Changlong Li, Jiamo Zhang

**Affiliations:** Department of Urology, Yongchuan Hospital, Chongqing Medical University, Chongqing, China

**Keywords:** malignant ureteral obstruction, allium, ureteral stenting, ureteroscopy, metal stent, endoscopic treatment

## Abstract

**Background:**

Malignant ureteral obstruction (MUO) has the potential to result in a range of outcomes, including varying degrees of hydronephrosis and renal impairment. Allium covered metal stents have provided a new, highly effective treatment option for MUO. Our objective was to evaluate the safety and efficacy of the Allium covered metallic stent for the treatment of malignant ureteral obstruction.

**Methods:**

Clinical data of 29 patients who underwent endoscopic insertion of an Allium ureteral stent between October 2019 and August 2021 at Yongchuan Hospital, affiliated with Chongqing Medical University, were analyzed retrospectively. At one, three, six and 12 months after insertion, serum creatinine was measured, the width of the renal pelvis was rechecked under ultrasound guidance, and the position and shape of the stent were checked by abdominal x-ray. Data on long-term stent patency, incidence of complications, changes in renal function and hydronephrosis grade were collected and analyzed. Ureteral stent symptom questionnaire (USSQ) was administered preoperatively and after six months in 22 patients with previous stent history.

**Results:**

Allium covered metal stents (*n* = 33) were implanted successfully in 29 patients who were followed for three to 22 months, with 32/33 stents remaining patent. Serum creatinine levels decreased in 17 patients (*p* < 0.001), and hydronephrosis decreased in 24 patients. Clavien-Dindo grade I complications were observed in seven patients, Clavien-Dindo II in one patient, whereas stent displacement and stent encrustation occurred in one patient each (Clavien-Dindo III–IV). USSQ scores had significantly improved six months after stent insertion (*p* < 0.001).

**Conclusions:**

Allium ureteral stents are a safe and effective treatment option for malignant ureteral obstruction, with good long-term patency, helping to preserve renal function and improve quality of life.

## Introduction

Malignant ureteral obstruction (MUO) is a common complication of malignant pelvic tumours. The cause of MUO is typically the direct invasion of the ureteral wall or the external compression of the ureter by a tumour. Additionally, periureteral or retroperitoneal fibrosis caused by adjuvant radiotherapy may also lead to or exacerbate ureteral obstruction. MUO results in varying degrees of hydronephrosis and impairment of renal function. Without effective treatment, MUO can cause a progressive decline in renal function, leading to renal failure and potentially life-threatening complications (1). Therefore, the prognosis and life expectancy of these patients remain poor. Previous studies have indicated that the median survival time for patients with MUO is between six and eight months, with an overall one-year survival rate below 50% (2).

The management and treatment of MUO present significant challenges. Surgical reconstruction and repair remain the gold standard for treating ureteral obstruction; however, in cases of MUO, the recurrence and failure rates are relatively high, and many patients are unable to undergo major surgery due to its associated risks (3).

Endoscopic techniques, with their operational simplicity, shorter surgical duration, minimal complications, safety, reliability, precise therapeutic outcomes, and rapid postoperative recovery, have become progressively and widely adopted in clinical practice. To a significant degree, they have supplanted surgical treatment and now represent a leading approach for managing MUO. As new materials have become available, various types of ureteral stents and nephrostomy tubes have been developed and are now widely used in the clinical management of MUO (4). Although minimally invasive and generally safe, these procedures are not without complications. Among them, iatrogenic ureteral strictures pose some of the greatest challenges in terms of timely diagnosis, effective treatment, and adequate follow-up. These strictures can be particularly harmful to patients, potentially resulting in partial or complete loss of kidney function. The incidence of ureteral stricture after ureteroscopy (URS) ranges from 0.3% to 4.9% (5). Moreover, a recent Delphi consensus highlighted that forgotten ureteral double-J stents are associated with an increased risk of stricture development. Stent-related complications, such as migration or encrustation, may further elevate the risk of ureteral stricture formation (6). However, complications associated with these materials, including gross hematuria, displacement, infection, and stenosis, can result in frequent postoperative replacement of ureteral stents or nephrostomy tubes for patients. Therefore, long-term placement of ureteral stents with effective drainage is essential for patients with MUO. In recent years, Allium covered metal stents have provided a new, highly effective treatment option for malignant ureteral stenosis with limited complications (7–9). This paper presents a retrospective analysis of 29 patients treated with Allium ureteral stents for MUO.

## Materials and methods

### Study design

In this retrospective study, we report data from 29 patients who underwent implantation of 33 Allium URS covered metal stents (Allium Medical, Israel) for MUO between October 2019 and August 2021 at Yongchuan Hospital. The study was approved by the Ethics Committee of Yongchuan Hospital Affiliated with Chongqing Medical University (reference 20210415), and was carried out according to the international ethical recommendations of the Declaration of Helsinki and its subsequent amendments and similar ethical standards. All participants were informed of the risks and benefits of stent implantation, and all patients consented to use the data for publication.

Inclusion criteria were defined as ureteral obstruction due to gastrointestinal, gynecological, or urological tumours, with a life expectancy of more than one year. Patients with obstruction by ureteral tumours and tumours of the renal pelvis were excluded.

All enrolled patients underwent preoperative urinalysis and urine culture. Patients with indications of urinary tract infection received antibiotics to control the infection. Surgical treatment was performed after reexamination confirmed the absence of infection in the urine. Patients without urinary tract infection receiveed preoperative prophylactic cefuroxime to prevent infection. Preoperative evaluation of stent-related symptoms using the USSQ was conducted in patients with a history of stent insertion (10, 11).

Malignant ureteral obstruction, its location, and the presence of hydronephrosis were confirmed by computed tomography (CT) scan. Additionally, the degree of hydronephrosis was graded as mild, moderate, or severe using ultrasound.

### Surgical procedure

The same skilled urologist performed all operations. Allium URS (URS-R-8-120) covered metal stents (AlliumMedical, Israel) were inserted retrogradely under fluoroscopic control.

Patients were brought under general anaesthesia and placed in the lithotomy position. Subsequently, an 8/9 Fr ureteroscope was placed, and the current stent on the affected side was pulled out of the external orifice of the urethra. A guidewire was retrogradely inserted via the end of the ureteral stent into the renal pelvis, and then the ureteral stent was removed entirely. The ureteroscope was then advanced into the ureter under the guidance of the guide wire up to the distal end of the stricture. After visualizing the location, length, and severity of the ureteral stricture by injecting diluted iohexol contrast solution into the ureter, the Budd balloon dilation catheter (18F or 21F, six centimetres) was retrogradely pushed along the guidewire to dilate the ureter for three to five minutes under a pressure of 25 Pa, until the “bee waist sign” of stricture disappeared.

**Figure 1:**
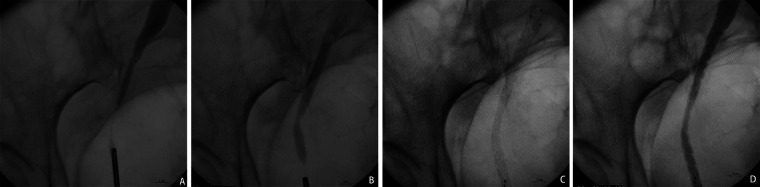
The surgical procedure for implanting Allium covered metal stent. **(A)** The retrograde pyelography under the ureteroscope shows the narrowed segment of the ureter. **(B)** The “bee waist sign” of the narrowed segment of the ureter is observed after the placement of the Budd balloon dilation catheter. **(C)** Allium covered metal stent is released and fully self-expanded and covers over the narrowed segment of the ureter. **(D)** Retrograde pyelography following release shows unobstructed drainage of the ureter.

The Allium URS ureteral stent was advanced over the guidewire until it covered the whole stricture segment. Then, the outer sheath of the stent was withdrawn until the stent was fully released and expanded. After slowly removing the delivery system, the position of the covered metal stent was verified again via X-ray, and adjustments to the stent position were made if necessary. Finally, the expansion of the covered metal stent was examined by retrograde pyelography and ureteroscopy (Figure 1).

### Data collection

Collected data included operation time, length of postoperative hospitalization, degree of hydronephrosis, change in serum creatinine after stent insertion, and the incidence of postoperative complications, graded according to the Clavien-Dindo scale (12).

Total operation time was defined as the time interval from insertion of the ureteroscope until completion of stent placement. Length of hospitalization was defined as the number of days from the day of the operation to the day of discharge from the hospital. Procedure failure was defined as increased hydronephrosis or deterioration of renal function due to stent migration, occlusion, or encrustation. Deterioration of renal function was defined as a doubling of serum creatinine levels, glomerular filtration rate (GFR) ≤50 mL/min or the need for dialysis treatment, or kidney transplantation. Stent patency was defined as stent implantation without evident migration, unanticipated stent exchange, or recurrent ureteral obstruction.

### Follow-up

Follow-up data were collected at one, three, six, and 12 months after discharge and consisted of determination of serum creatinine, ultrasound evaluation of renal pelvis width, abdominal X-ray to evaluate stent shape and position, and clinical evaluation. Evaluation of stent-related symptoms using the USSQ was performed six months after insertion in patients with a history of prior stent insertion.

In the event that the displaced stent fails to cover the stenotic segment, it is necessary to adjust the stent position ureteroscopically.

### Statistics

Numerical data following a normal distribution were expressed as mean ± standard deviation and were analyzed using a paired samples *t*-test. Not normally distributed parameters were expressed as median and range. Categorical variables were presented as frequency or percentage. A *p*-value <0.05 was considered statistically significant. SPSS 23.0 software was used for statistical analysis.

## Results

The baseline characteristics of the 29 patients before stent insertion are presented in Table 1. Twenty-four patients were pathologically confirmed as malignant tumours after tumour resection or biopsy, while the remaining five were clinically diagnosed. All 29 patients had tumour stages ranging from Ⅲ to Ⅳ. Twenty-two patients had a history of indwelling or repeated replacement of ordinary ureteral stents for over six months, of which six cases began to develop hydronephrosis while having an indwelling ureteral stent.

**Table 1:** General patient characteristics before stent insertion.

Patient characteristics	
Number of patients	29
Number of implanted stents	33
Age, years (mean ± SD)	61 ± 13
Gender (male/female)	10/19
Prior history of stenting (*n*, %)	22 (75%)
Etiology of malignant obstruction	
Cervical cancer	10 (35%)
Ovarian cancer	6 (21%)
Rectal cancer	8 (28%)
Sigmoid colon cancer	2 (7%)
Posterior peritoneal tumour	2 (7%)
Prostate cancer	1 (3%)
Ureteral stricture characteristics	
Length [cm, median (range)]	3.6 (2.0–8.6)
Stricture location (*n*, %)	
Upper ureter	2 (7%)
Middle ureter	18 (62%)
Lower ureter	13 (45%)
Hydronephrosis grade (*n*, %)	
Mild	12 (36%)
Moderate	15 (46%)
Severe	6 (18%)

In the study population, 33 Allium URS stents were successfully inserted retrogradely, with unilateral insertion in 25 patients and bilateral insertion in four. The median length of the ureteral stricture was 3.6 cm (range of 2.0–8.6 cm) (Table 1). The median operation time was 50.2 min, and the median hospitalization duration was four days.

### Complication

No serious perioperative complications were observed, but mild perioperative complications of Clavien-Dindo grade I occurred in seven patients (21%) (Table 2). All of them were resolved with conservative treatment. During the follow-up period, one patient (3%) experienced recurrent urinary tract infection requiring antibiotic treatment (Clavien-Dindo grade II). Stent migration (3%) and encrustation (3%) (Clavien-Dindo grades III–IV) each occurred in one ureter (Table 2). The stent migration was treated with reimplantation after stent extraction, while the encrusted stent, due to stone formation, was removed, and the ureteral obstruction treated by nephrostomy.

**Table 2:** Operation characteristics and complication rates graded according to the Clavien-Dindo scale.

Variable	Value
Operative time, median (range)	50.2 min (30–140)
Length of hospital stay, median (range)	4 days (3–7)
Follow up, median (range)	10 months (3–22)
Stent patency (%)	32 (97%)
Postoperative complications	*n* (%)
Clavien Dindo grade I	
Persistent pain	2 (6%)
Haematuria after activity	3 (9%)
Lower urinary tract symptoms	2 (6%)
Clavien Dindo grade II	
Infection requiring antibiotics	1 (3%)
Clavien Dindo grade III–IV	
Stent migration	1 (3%)
Stent encrustation	1 (3%)

### Effectiveness

Serum creatinine levels significantly decreased in 17 patients (*p* < 0.001). Serum creatinine levels remained normal postoperatively and during follow-up in 12 patients who had normal levels preoperatively. In nine patients with preoperative serum creatinine levels above 300 μMol/L, levels returned to normal three months after stent insertion. In eight patients with preoperative serum creatinine levels above 200 μMol/L, levels returned to normal one month after stent insertion. Furthermore, hydronephrosis was reduced to varying degrees in 24 treated ureters.

### Follow-up

**Figure 2:**
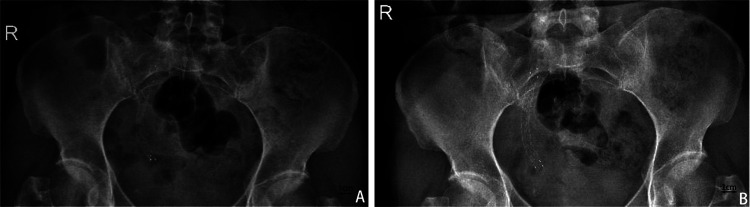
Abdominal X-ray to evaluate stent shape and position. **(A)** One month after the placement of Allium covered metal stent. **(B)** X-ray one year after the placement of Allium covered metal stent shows more complete stent release.

Follow-up duration ranged from three to 22 months with a median of 10 months. Stent patency rate was 97%, with 32/33 stents remaining functional without evidence of migration or recurrence of obstruction throughout the follow-up period (Table 2). The stent demonstrated a more satisfactory expansion effect as time progresses (Figure 2). None of the treated patients requested to remove the stent.

### Living quality

In 22 patients with a history of prior urinary stent insertion, significantly lower USSQ subgroup scores six months after Allium stent insertion indicated substantial improvements in urinary symptoms, physical pain, overall health status, work performance and additional problems compared to the preoperative situation (*p* < 0.05, Table 3). Most of the patients in the study were no longer sexually active due to age or comorbidities; therefore, the sexual matters component of the USSQ was not analyzed. Patient satisfaction with the stent tended to increase over time in some patients.

**Table 3:** USSQ scores after insertion of the Allium stent in patients with a previous history of indwelling stents.

USSQ subgroup	Baseline^a^	6 months	*t*	*p*
Urinary symptoms	31.1 ± 2.9	20.4 ± 2.1	14.1	<0.001
Body pain	18.5 ± 2.6	9.5 ± 1.2	13.8	<0.001
General health	14.3 ± 2.0	9.1 ± 1.7	9.2	<0.001
Work performance	11.1 ± 2.1	5.5 ± 2.0	10.4	<0.001
Sexual matters	–	–	–	–
Additional problems	12.6 ± 2.4	7.5 ± 1.6	12.3	<0.001

^a^ USSQ scores are expressed as mean ± SD. Scores at baseline and after 6-months were compared using a paired *t*-test.

## Discussion

The incidence of MUO is on the rise. Ureteral obstruction can be intrinsic or extrinsic (13). Common extrinsic causes include ovarian, cervical, colon, sigmoid, prostate, and other pelvic malignancies (14, 15). Treatment of these tumours with chemotherapy and radiotherapy can result in retroperitoneal fibrosis, which can exacerbate the obstruction (14). Effectively relieving ureteral obstruction is crucial for both prolonging survival and improving the quality of life for patients (15). Most patients with MUO have a history of abdominal surgery or radiotherapy and develop ureteral obstruction at advanced stages of tumour progression, when their physical condition is poor and life expectancy is limited. Since complete removal of ureteral obstruction is impossible in most of these patients, intraluminal retrograde ureteral stent placement has become the preferred method to relieve obstruction and protect renal function (16).

Single polymer ureteral stent placement was the initial method for treating MUO. Success rates of retrograde ureteral stents have steadily improved over the years; even so, failure rates have been reported to be as high as 40% with traditional single polymeric ureteral stents (17). In addition to primary tumor progression, stent occlusion due to urine viscosity, aggregation of fragments and macromolecules, as well as external compressive forces, mucus production, and urothelial sloughing, frequently cause stent failure (18, 19). Traditional ureteral stents made of silicone resin or polyurethane have a soft texture and small lumen, resulting in low compressive capacity and a tendency for blockage. These stents can induce stones, cause catheter-related infections, and frequently lead to stent-related symptoms that significantly affect the quality of life of patients (20–22). To address these limitations, frequent stent replacement every three months is recommended. This approach imposes a tremendous economic burden and considerable physical and psychological strain on patients (23). Moreover, this practice increases the risk of complications, including ureteral stricture.

Metal stents offer advantages for treating MUO, including high-pressure resistance, tensile strength, flexibility, and strong corrosion resistance (24, 25). Liatsikos et al. reported 100% effectiveness of urinary drainage in 25 patients with MUO treated with the Resonance metallic stent without stent displacement during the 11-month follow-up period. Approximately half of the patients required a stent exchange after a mean indwelling period of 11 months (8–14 months). Additionally, about 59% developed urinary tract infections and/or lower urinary tract irritation (26). The recommended retention period for metal stents is 12 months. However, while replacement of the Resonance stent after one year was not necessary for 70%–80% of patients with MUO, some patients with rapid tumor progression or frequent stone formation required replacement at intervals shorter than one year (27). The effectiveness of obstruction relief in MUO is determined by both stent stiffness and ability to withstand external pressure, as well as the effective stent diameter. These factors have been shown to reduce the stent failure rate and delay the need for percutaneous nephrostomy (28).

In recent years, accumulating evidence indicates that Allium self-expanding fully covered metal stents are effective for treating both neoplastic and benign ureteral strictures (8, 9). These stents are large-diameter segmental devices available in various lengths and diameters, designed to bridge only the strictured or obstructed segment of the ureter, without pigtail curls extending into the renal pelvis or bladder. Allium stents consist of a metal skeleton made of nickel-titanium memory alloy fully covered by a copolymer coating. The titanium alloy core ensures elasticity and strong resistance to radial forces to maintain ureter patency in the presence of compressive forces, whereas the polyurethane surface cover prevents tissue ingrowth, resulting in long retention times, strong compressive capacity, sufficient drainage, easy removal, good comfort, low infection rate, and no obvious lower urinary tract symptoms compared with other ureteral stents (7–9). Moskovitz et al. reported successful insertion of Allium stents in 48 of 49 patients, achieving 100% effective obstruction relief during an average follow-up period of 21 months (29). Stent-related symptoms were significantly reduced, and no patients required stent removal due to complications. The main complication was stent displacement in seven (10%) ureters, with migration to the bladder in five cases and migration to the renal pelvis in two cases. Wang et al. reported that stent patency remained over a median follow-up time of 16 months (eight to 21 months) in 27/32 patients treated with an Allium stent for MUO after surgery or radiotherapy (9). In this study, stent displacement was observed in four patients within 10 months after implantation, and obstruction occurred in three stents. A large-scale prospective study with 147 patients receiving 157 Allium stents reported effective urinary drainage in nearly all patients. The most common complications were hematuria (13 cases, 8.8%), followed by urinary tract infection (11 cases, 7.5%) and pain (8 cases, 5.4%), and distal ureteral stenosis was found to be an independent predictor of stent failure (30). Gao et al. reported a success rate of 92% in 25 patients followed for a median of 12 months after Allium stent insertion, with 16% reporting pain and four percent having fever after stent insertion. They also observed decreased hydronephrosis volume and blood urea nitrogen levels after stent insertion (31). A global multicenter study of 92 patients reported that after a mean follow-up of 27 months (7), 1.1 percent (one patient) developed stent obstruction, and 18 patients had their stents removed as scheduled after one year of retention and were asymptomatic for 59 months of follow-up.

In line with the literature cited above, our study also demonstrated that Allium stents are effective for the treatment of MUO, with high patency rates, reduced hydronephrosis and protection of renal function. In our study, the incidence of urinary tract infection was three percent, with stent migration and encrustation also occurring at a rate of three percent. Additionally, serum creatinine levels decreased in 59% of patients and remained normal in 41%, suggesting that Allium stents offer stable long-term drainage, maintain patency, promptly relieve obstruction, and protect renal function. Compared with non metal stents, metal stents provide superior patency rates and comfort, with fewer complications and lower failure rates. Furthermore, we also found that patient satisfaction and tolerance of the stent improved over time, and that stent-related symptoms were significantly decreased after six months in patients with a history of prior stenting across all investigated USSQ subscores. Allium stents are registered for a retention period of up to three years, with reports of patients who have retained them for up to 10 years. As the majority of MUO patients survive for less than three years, it is challenging to determine the necessity for stent removal. However, existing studies suggest a correlation between stent-related complications and the length of time the stent is retained. Consequently, stent replacement may be a viable option in cases of infection, crusting, obstruction, and displacement. While some non metal stents can last for six to 12 months, their small lumen limits their compressive capacity, making them prone to blockage and less suitable for long-term drainage. The patency rate of Allium stents was significantly better than that of non metal stents during a mean follow-up period of one year. Relief of ureteral obstruction by Allium or Resonance stents has been shown to be equally effective. However, the USSQ score, incidence of recurrent urinary tract infections, and prevalence of pain and discomfort were significantly higher in the Resonance group compared to the Allium group. Conversely, Allium stents exhibited a higher rate of reobstruction (32). A number of studies have shown that for benign strictures, the ureter can remain unobstructed for a long time after stent removal, suggesting that stent placement may effectively resolve the stricture.

### Limitation

However, several limitations regarding our study warrant discussion. First, clinical data were collected in a retrospective manner and results may be limited by unknown confounding factors. Second, the relatively small sample size, due to the limited number of patients with the disease, may affect the statistical significance of the results. Third, there was no control group in this study, making it difficult to eliminate the interference and influence of non-experimental factors.

The heterogeneity of patient populations, diversity of practice, and lack of direct comparative studies complicate the assessment of the effectiveness of different ureteral stents for MUO. Larger-scale, prospective and randomized clinical trials involving these techniques are necessary to help provide evidence-based medicine and guidelines for MUO management. Urologists need to tailor treatment to the patient to ensure effectiveness while preserving the quality of life.

## Conclusion

This study indicates that Allium ureteral stents are both effective and safe for the treatment of MUO. They maintain relatively long-term patency rates, help to preserve renal function, and improve patients' quality of life.

## Data Availability

The original contributions presented in the study are included in the article/Supplementary Material, further inquiries can be directed to the corresponding author.

## References

[1] Khoo CC Abboudi H Cartwright R El-Husseiny T Dasgupta R. Metallic ureteric stents in malignant ureteric obstruction: a systematic review. Urology. (2018) 118:12–20. 10.1016/j.urology.2018.01.019 29408390

[2] O’Connor EM Nason GJ Kiely EA. Urological management of extramural malignant ureteric obstruction: a survey of Irish urologists. Curr Urol. (2017) 11(1):21–5. 10.1159/000447190 29463973 PMC5814774

[3] Cordeiro MD Coelho RF Chade DC Pessoa RR Chaib MS Colombo-Júnior JR A prognostic model for survival after palliative urinary diversion for malignant ureteric obstruction: a prospective study of 208 patients. BJU Int. (2015) 117(2):266–71. 10.1111/bju.12963 25327474

[4] Alma E Ercil H Vuruskan E Altunkol A Unal U Gurlen G Long-term follow-up results and complications in cancer patients with persistent nephrostomy due to malignant ureteral obstruction. Support Care Cancer. (2020) 28(11):5581–8. 10.1007/s00520-020-05662-z 32757161

[5] Moretto S Saita A Scoffone CM Talso M Somani BK Traxer O Ureteral stricture rate after endoscopic treatments for urolithiasis and related risk factors: systematic review and meta-analysis. World J Urol. (2024) 42(1):234. 10.1007/s00345-024-04933-2 38613692

[6] Moretto S Saita A Scoffone CM Talso M Somani BK Traxer O An international Delphi survey and consensus meeting to define the risk factors for ureteral stricture after endoscopic treatment for urolithiasis. World J Urol. (2024) 42(1):412. 10.1007/s00345-024-05103-0 39002090

[7] Bahouth Z Meyer G Halachmi S Nativ O Moskowitz B. Multicenter experience with allium ureteral stent for the treatment of ureteral stricture and fistula. Harefuah. (2015) 154(12):753–6, 806. 26897774

[8] Bahouth Z Moskovitz B Halachmi S Nativ O. Allium stents: a novel solution for the management of upper and lower urinary tract strictures. Rambam Maimonides Med J. (2017) 8(4):e0043. 10.5041/RMMJ.10313 28872453 PMC5652934

[9] Wang W Gao X Chen J Liu Z Peng L Wei X. Metal stent for the ureteral stricture after surgery and/or radiation treatment for malignancy. BMC Urol. (2021) 21(1):146. 10.1186/s12894-021-00912-6 34656100 PMC8520268

[10] Joshi HB Newns N Stainthorpe A MacDonagh RP Keeley FX Timoney AG. Ureteral stent symptom questionnaire: development and validation of a multidimensional quality of life measure. J Urol. (2003) 169(3):1060–4. 10.1097/01.ju.0000049198.53424.1d 12576846

[11] Zhu C Qu J Yang L Feng X. The Chinese linguistic validation of the ureteral stent symptom questionnaire. Urol Int. (2019) 102(2):194–8. 10.1159/000493764 30368512

[12] Dindo D Demartines N Clavien P-A. Classification of surgical complications. Ann Surg. (2004) 240(2):205–13. 10.1097/01.sla.0000133083.54934.ae 15273542 PMC1360123

[13] Vorobev V Beloborodov V Golub I Frolov A Kelchevskaya E Tsoktoev D Urinary system iatrogenic injuries: problem review. Urol Int. (2021) 105(5–6):460–9. 10.1159/000512882 33535218

[14] Song Y Fei X Song Y. Percutaneous nephrostomy versus indwelling ureteral stent in the management of gynecological malignancies. Int J Gynecol Cancer. (2012) 22(4):697–702. 10.1097/IGC.0b013e318243b475 22315095

[15] Fokdal L Tanderup K Pötter R Sturdza A Kirchheiner K Chargari C Risk factors for ureteral stricture after radiochemotherapy including image guided adaptive brachytherapy in cervical cancer: results from the EMBRACE studies. Int J Radiat Oncol Biol Phys. (2019) 103(4):887–94. 10.1016/j.ijrobp.2018.11.006 30419309

[16] Shoshany O Erlich T Golan S Kleinmann N Baniel J Rosenzweig B Ureteric stent versus percutaneous nephrostomy for acute ureteral obstruction—clinical outcome and quality of life: a bi-center prospective study. BMC Urol. (2019) 19(1):79. 10.1186/s12894-019-0510-4 31455309 PMC6712738

[17] Tabib C Nethala D Kozel Z Okeke Z. Management and treatment options when facing malignant ureteral obstruction. Int J Urol. (2020) 27(7):591–8. 10.1111/iju.14235 32253785

[18] Shilo Y Modai J Leibovici D Dror I Berkowitz B. The impact of ureteral deformation and external ureteral pressure on stent failure in extrinsic ureteral obstruction: an *in vitro* experimental study. J Endourol. (2020) 34(1):68–73. 10.1089/end.2019.0465 31359787

[19] Karthikeyan VS Kim SH Park B Joo J Joung JY Seo HK Retrograde pyelography predicts retrograde ureteral stenting failure and reduces unnecessary stenting trials in patients with advanced non-urological malignant ureteral obstruction. PLoS One. (2017) 12(9):e0184965. 10.1371/journal.pone.0184965 28931043 PMC5607161

[20] Zhang J-M Liu J Wang K Zhang X Zhao T Luo H-M. Observations of bacterial biofilm on ureteral stent and studies on the distribution of pathogenic bacteria and drug resistance. Urol Int. (2018) 101(3):320–6. 10.1159/000490621 30212821

[21] Lu J Lu Y Xun Y Chen F Wang S Cao S. Impact of endourological procedures with or without double-J stent on sexual function: a systematic review and meta-analysis. BMC Urol. (2020) 20(1):13. 10.1186/s12894-020-0582-1 32059655 PMC7023811

[22] Vogt B. Ureteral stent obstruction and stent’s discomfort are not irreparable damages. Urol Case Rep. (2018) 20:100–1. 10.1016/j.eucr.2018.07.025 30101077 PMC6076363

[23] Yossepowitch O Lifshitz D Dekel Y Gross M Keidar D Neuman M Predicting the success of retrograde stenting for managing ureteral obstruction. (2001) (5):1746–9. 11586215

[24] Chen Y Liu C Zhang Z Xu P Chen D Fan X Malignant ureteral obstruction: experience and comparative analysis of metallic versus ordinary polymer ureteral stents. World J Surg Oncol. (2019) 17(1):74. 10.1186/s12957-019-1608-6 31039812 PMC6492337

[25] Kang Q Jiang F Yu Y Yang B. Application of metallic ureteral stents in gynecological malignancies: a literature review. Minim Invasive Ther Allied Technol. (2019) 29(1):1–9. 10.1080/13645706.2019.1572626 30793634

[26] Liatsikos E Kallidonis P Kyriazis I Constantinidis C Hendlin K Stolzenburg J-U Ureteral obstruction: is the full metallic double-pigtail stent the way to go? Eur Urol. (2010) 57(3):480–7. 10.1016/j.eururo.2009.02.004 19232816

[27] Miyazaki J Onozawa M Takahashi S Maekawa Y Yasuda M Wada K The resonance® metallic ureteral stent in the treatment of malignant ureteral obstruction: a prospective observational study. BMC Urol. (2019) 19(1):137. 10.1186/s12894-019-0569-y 31881875 PMC6935232

[28] Haifler M Kleinmann N Weiss D. Tandem ureteral stents drainage lowers renal pelvis pressure in malignant ureteral obstruction: experimental and computational models. J Biomech. (2021) 117:110237. 10.1016/j.jbiomech.2021.110237 33486265

[29] Moskovitz B Halachmi S Nativ O. A new self-expanding, large-caliber ureteral stent: results of a multicenter experience. J Endourol. (2012) 26(11):1523–7. 10.1089/end.2012.0279 22697886

[30] Gao X Song T Peng L Yuan C Wang W Chen J Self-expanding metal ureteral stent for ureteral stricture: experience of a large-scale prospective study from a high-volume center—cross-sectional study. Int J Surg. (2021) 95:106161. 10.1016/j.ijsu.2021.106161 34728417

[31] Gao X Chen J Wang W Peng L Di X Xiao K Step-by-step technique for the endoscopic treatment of ureteric stricture. BJU Int. (2021) 128(6):692–6. 10.1111/bju.15558 34322987

[32] Gao W Xing T Ou T. The resonance and the allium ureteral stents in the treatment of non-malignant refractory ureterostenosis. BMC Urol. (2021) 21(1):53. 10.1186/s12894-021-00815-6 33827529 PMC8025479

